# Extending radiowave frequency detection range with dressed states of solid-state spin ensembles

**DOI:** 10.1038/s41534-024-00891-0

**Published:** 2024-10-26

**Authors:** Jens C. Hermann, Roberto Rizzato, Fleming Bruckmaier, Robin D. Allert, Aharon Blank, Dominik B. Bucher

**Affiliations:** 1 Technical University of Munich, TUM School of Natural Sciences, Department of Chemistry, Lichtenbergstraße 4, Garching bei München, 85748 Germany; 2 Munich Center for Quantum Science and Technology (MCQST), Schellingstr. 4, München, 80799 Germany; 3 QuantumDiamonds GmbH, Friedenstr. 6, München, 81671 Germany; 4 Schulich Faculty of Chemistry, Technion - Israel Institute of Technology, Haifa, 32000 Israel

**Keywords:** Quantum metrology, Qubits

## Abstract

Quantum sensors using solid-state spin defects excel in the detection of radiofrequency (RF) fields, serving various applications in communication, ranging, and sensing. For this purpose, pulsed dynamical decoupling (PDD) protocols are typically applied, which enhance sensitivity to RF signals. However, these methods are limited to frequencies of a few megahertz, which poses a challenge for sensing higher frequencies. We introduce an alternative approach based on a continuous dynamical decoupling (CDD) scheme involving dressed states of nitrogen vacancy (NV) ensemble spins driven within a microwave resonator. We compare the CDD methods to established PDD protocols and demonstrate the detection of RF signals up to ~85 MHz, about ten times the current limit imposed by the PDD approach under identical conditions. Implementing the CDD method in a heterodyne/synchronized protocol combines the high-frequency detection with high spectral resolution. This advancement extends to various domains requiring detection in the high frequency (HF) and very high frequency (VHF) ranges of the RF spectrum, including spin sensor-based magnetic resonance spectroscopy at high magnetic fields.

## Introduction

The detection of weak radiofrequency (RF) magnetic fields by spin-based magnetometers has been shown to be of importance for many applications, ranging from fundamental physics^1,2^, communications^3,4^, and chemical analysis^5–9^. In particular, quantum sensors based on ensembles of spin defects in solid-state systems have shown increased sensitivity^5,10–14^ and imaging capabilities^15,16^ in detecting weak signals, even from nuclear spins in micro- and nanoscale sample volumes. Typical sensing protocols rely on pulsed dynamical decoupling (PDD) methods that effectively decouple the sensor spins from the environmental noise and increase the sensitivity to RF signals. Specifically, PDD methods use a train of microwave (MW) pulses, where the time intervals between pulses must be precisely matched to the RF frequency of interest^17–26^. Although these sequences can significantly extend spin coherence times and provide high sensitivity for detecting low-frequency signals (sub-MHz to a few MHz), they ultimately fail when higher frequencies need to be detected^26–29^. This is primarily constrained by the finite MW pulse width, determined ultimately by the available MW power^30–32^. In addition, the use of a large number of MW pulses can lead to a significant accumulation of errors due to pulse imperfections^22,33–36^. Finally, from a technological perspective, the sampling rates of the waveform generators used to synthesize the microwave pulses can also be a practical limitation. All of these factors represent significant limitations, especially in applications that require the detection of higher RF frequencies, such as in the case of spin sensor-based nuclear magnetic resonance (NMR) spectroscopy. In this context, the use of stronger bias magnetic fields to improve intrinsic spectral resolution requires the detection of higher nuclear Larmor frequencies^31,37,38^.

Continuous dynamical decoupling (CDD) methods, such as spinlock (SL)—based sequences, detect RF fields by adjusting their MW amplitude rather than the MW pulse spacing, and offer a potential solution to these problems^28,39–43^. Furthermore, the sensitivity of spinlock-based methods relies on the longitudinal relaxation time in the rotating frame (*T*_1*ρ*_), which is typically much longer than the spin coherence time (*T*_2_) that limits pulsed experiments^28,44–46^. However, SL protocols necessitate precise control over the MW field amplitude and strong and spatially homogeneous microwave (MW) fields are particularly needed for NV-ensembles^29,45^. Thus, it is crucial to achieve high MW field strengths to match the Rabi frequency with the RF signals and ensure high spatial homogeneity of the MW drive for effective spinlock pulses over the spin defect ensemble. This makes the optimization of MW delivery critically important for the successful performance of CCD experiments.

In this work, we use an ensemble of nitrogen-vacancy (NV) centers in diamond and a resonant MW structure operating at ~9.4 GHz (X-band) to demonstrate the detection of RF signals at frequencies up to ~85 MHz, approximately one order of magnitude higher than the frequency detection limit imposed by PDD experiments under the same experimental conditions. Finally, we exploit the phase sensitivity inherent in the spin locking protocol to implement a novel CDD-based coherently averaged synchronized readout (CDD-CASR) detection scheme^7,28,47,48^. This method allows the detection of RF signals with high spectral resolution and, with our modification, extends the detectable frequency range by an order of magnitude^8,9^.

## Results

### Theoretical background

**Fig. 1:**
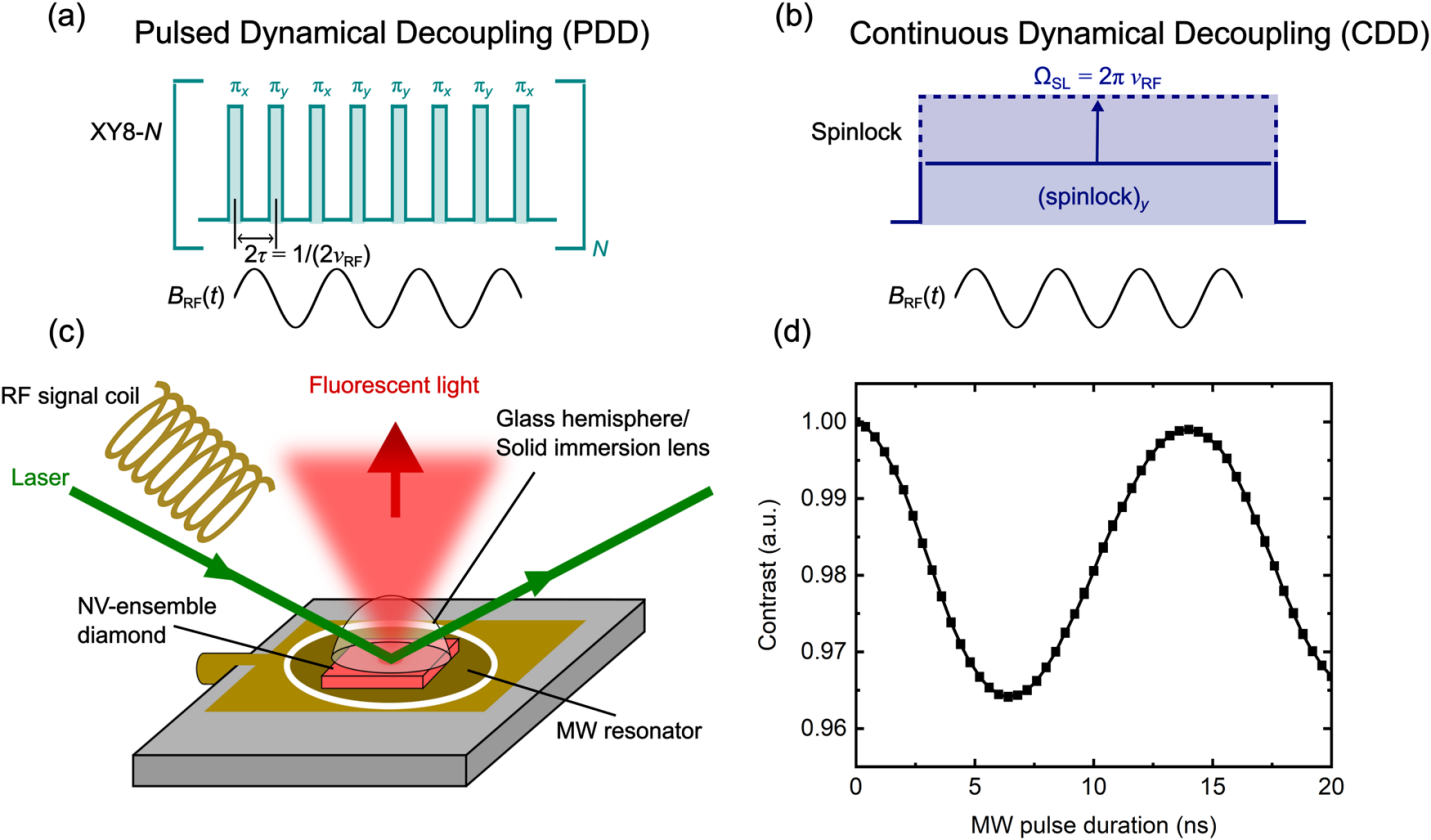
RF sensing with ensembles of NV-centers in a MW structure. **a** In conventional Pulsed Dynamical Decoupling (PDD) sequences, such as XY8-*N*, the intervals between *π* pulses must align with the period of the sensed RF. This requirement restricts their sensing capabilities, primarily due to pulse overlap and pulse errors. **b** In contrast, Continuous Dynamical Decoupling (CDD) sequences, such as the spinlock scheme, match the spinlock pulse amplitude to the sensing frequency, limiting their maximum detectable frequency to the highest achievable microwave amplitude, *Ω*_SL_. **c** The experimental setup consists of a dielectric resonator with an NV diamond chip placed inside its cavity. This setup allows for high-power MW pulsing with a homogeneous magnetic field distribution over the NV ensemble area, making it an ideal platform for RF sensing using DD schemes. **d** With the assembly in (**c**), Rabi frequencies up to ~85 MHz can be achieved, corresponding to *π*-pulse durations of ~6 ns.

In most quantum sensing schemes, the spin states of the NV centers are first initialized to the $\left\vert 0\right\rangle$ state by a laser pulse and then transferred to a superposition state by a *π*/2 MW pulse. In this sensing state, alternating magnetic fields can be detected by selective manipulation of the spin state via MW pulses using pulsed (PDD) or continuous dynamical decoupling (CDD) schemes. PDD methods used in this work, such as the XY8-*N* sequence, consist of *N* repetitions of a block of eight *π* pulses around the *x*- and *y*-axis (see Fig. 1a). In these conditions, a relative phase ${\theta }_{{\rm{PDD}}}({t}_{s})=(2/\pi )\gamma {\hat{B}}_{{\rm{RF}}}{t}_{s}$ between the $\left\vert 0\right\rangle$ and $\left\vert 1\right\rangle$ states is accumulated only when the matching condition *ν*_RF_ = 1/(4*τ*) between the RF signal frequency *ν*_RF_ and the spacing 2*τ* between the *π* pulses is met. Here, *γ* is the gyromagnetic ratio of the electron, ${\hat{B}}_{{\rm{RF}}}$ the RF magnetic field amplitude and *t*_*s*_ = 16*Nτ* the interrogation time during which the phase *θ* is accumulated. To date, this has been the method of choice in NV-based quantum sensing, as it provides optimal sensitivities for signals up to a few MHz frequency and is particularly robust to pulse imperfections due to the alternate pulse phase design^11,15,16,21,26,49–51^.

Differently, the CDD sensing method relies on applying a long pulse along the *y*-axis (see Fig. 1b) immediately after generating electron spin coherence with a strong (*π*/2)_*x*_ pulse. This effectively locks the spins onto the equatorial plane of the Bloch sphere in the rotating frame (spinlock (SL)). In this protocol, the NV spins are brought into their dressed states (DS), where an artificial low-field condition is created and their pseudo-Zeeman splitting is determined by the Rabi frequencies of the bare states^52^. The lifetime of such a spin-locked state is limited by the spin-lattice relaxation in the rotating frame *T*_1*ρ*_ which is typically longer than the decoherence times (*T*_2_). This technique has been extensively used in NMR spectroscopy and in Dynamic Nuclear Polarization (DNP) experiments, where it enables polarization transfer between different nuclear spins, e.g. ^1^H and ^13^C^39,53^ and between spins at very different energy splittings, such as electron and nuclear spins^54–56^. In addition, an arbitrary RF field with polarization transverse to the quantization axis of the dressed states can drive transitions between them and induce a rotating frame Rabi nutation whose dynamics encodes information about the sensed RF field^28,40,41^ (see Supplementary Note 1 in the Supplementary Information). In particular, during the SL time *t*_*s*_, a phase ${\theta }_{{\rm{SL}}}=\frac{1}{2}\gamma {\hat{B}}_{{\rm{RF}}}{t}_{s}$ can be accumulated if the matching condition *Ω*_SL_ = 2*πν*_RF_ is fulfilled. This pulse sequence has been successfully demonstrated with NV-centers in diamond and other spin defects in alternative materials^26,28,41,45,46,57^.

### Microwave delivery

The experiment is based on a 1 mm × 1 mm × 0.5 mm electronic grade single crystal diamond chip on which an NV center-doped 10 μm layer with 10 ppm substitutional nitrogen (P1 centers) was homoepitaxially grown (see Methods for further details). In order to achieve a strong and homogenous MW drive over the NV ensemble, we designed a single-sided dielectric resonator operating at a frequency of ~9.4 GHz (X-band), which is open on one side (see Fig. 1c, Methods and Supplementary Note 2 in the Supplementary Information)^58^, in which the diamond can be inserted. This enables us to reach high Rabi frequencies of up to ~85 MHz and corresponding *π*-pulse durations of ~6 ns (see Fig. 1d and Supplementary Note 3). In contrast, typical achievable Rabi frequencies are on the order of 20 MHz with strong spatial inhomogeneities when the MW is delivered using simple microwave loops positioned on the NV-diamond surface^13,45,59^. The assembly is positioned in the center of an electromagnet operating at a magnetic field strength *B*_0_ of ~0.2 T. This device combines precise MW pulsing, high-power MW generation and homogeneity, and uniform magnetic field distribution over the NV-ensemble. With this apparatus, we conduct RF sensing using a CDD protocol and compare its performance with a conventional PDD method based on an XY8-*N* sequence.

### Testing the RF frequency response

**Fig. 2:**
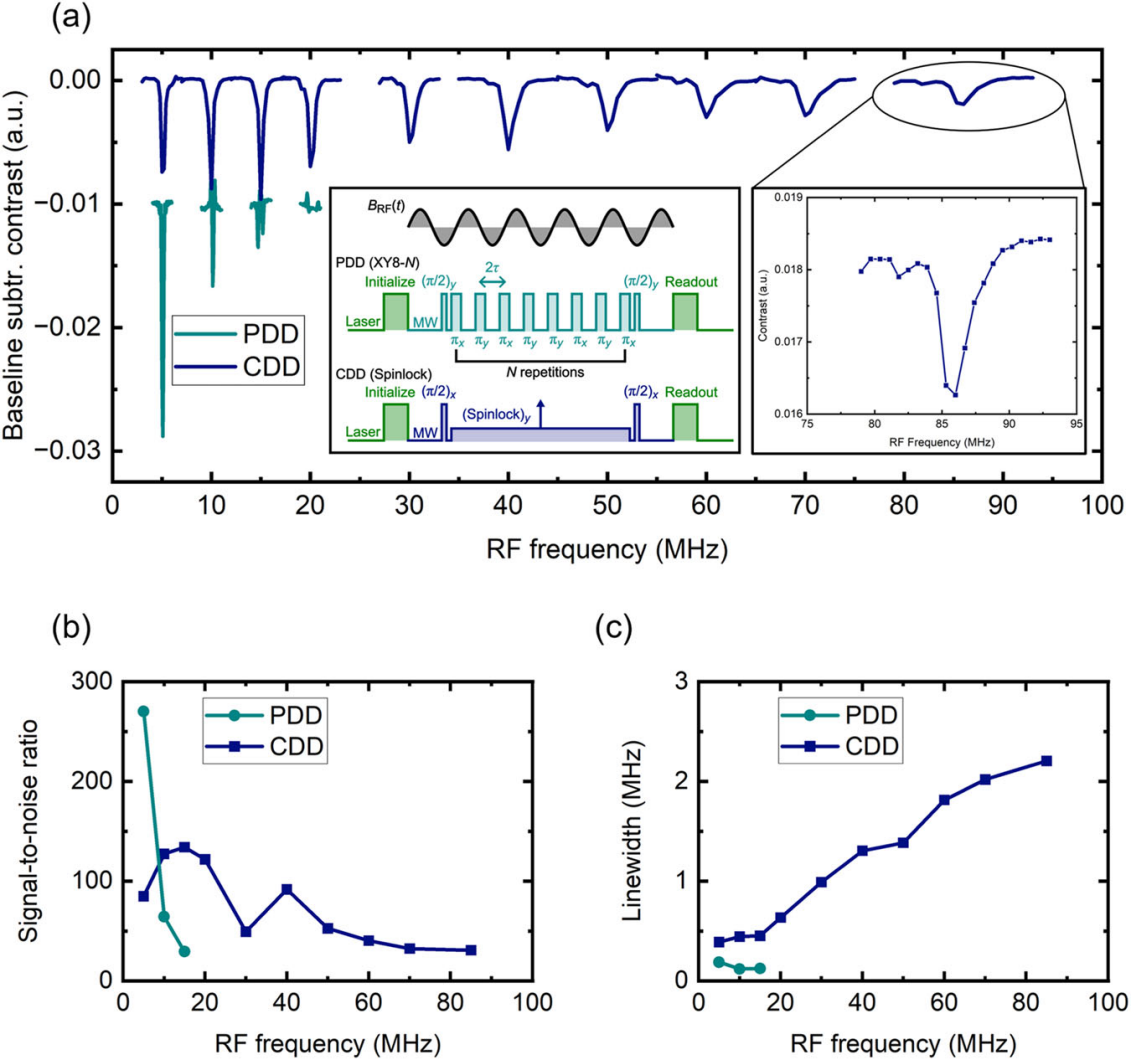
Extending the RF sensing range of NV ensemble based sensors. **a** Measured frequency responses of PDD (XY8-*N*) and CDD (spinlock) protocols (left inset) to an external RF field at different *ν*_RF_. The pulse spacing 2*τ* in case of PDD and the amplitude *Ω*_SL_ of the spinlock pulse for the CDD are kept constant while the RF frequency is swept. Each dip corresponds to a single matching condition. The sensing limit (~10 MHz) of the PDD scheme can be overcome by CDD, ultimately achieving a maximum sensing frequency of ~85 MHz (right inset). Signal-to-noise ratio (SNR) (**b**) and linewidths (**c**) of the corresponding data in (**a**).

In our first set of experiments, we test the maximum frequency *ν*_RF_ of an RF field that the PDD and CDD protocols can detect. RF signals are generated by an antenna placed next to the diamond, and their frequency is measured by observing a dip in the NV’s fluorescence contrast when the phase accumulation condition is met, as described above. We set a constant pulse spacing in PDD and a constant spinlock amplitude in the CDD case and keep the phase accumulation time the same for both experiments. By sweeping the applied RF frequency (see Fig. 2a, inset), the frequency response of the NV sensor can be probed.

Figure 2a shows the sensing of RF frequencies using an XY8-*N* pulse sequence. The detected signal intensity decreases rapidly with increasing frequency *ν*_RF_ and disappears ultimately around 20 MHz. We examined the pulse sequence in the time domain after the resonator, which reveals that the MW pulses are distorted and exhibit a tail of approximately 25 ns following the end of each pulse due to the MW ringing from the resonator (see Supplementary Note 4 in the Supplementary Information). This results in a pulse overlap for *τ*-spacings on the order of 30 ns, which is consistent with degrading sensing capabilities for frequencies above 8 MHz. This demonstrates the difficulties with detecting high frequencies with PDD. In contrast, using the CDD protocol, signals ≳ 85 MHz are successfully detected. However, their signal-to-noise ratio (SNR) is three times lower than that of the XY8-*N* experiments at low frequencies, when considering the peak intensity, with the noise remaining at a comparable magnitude for both pulse sequences (see Fig. 2b). The integral under the peaks is comparable in both cases, indicating that the signal is distributed over a wider linewidth in the CDD case. This can be attributed to the microwave (MW) field inhomogeneity over the NV ensemble region, which is still suboptimal in our experimental setting. The trends in the linewidths are shown in Fig. 2a, c). The recorded XY8-*N* dips show narrower linewidths compared to the spinlock counterparts, although accurate linewidth estimation for signals recorded at frequencies >5 MHz is challenging due to significant signal distortion. Nevertheless, it is apparent that, in the case of CDD, the linewidth gradually increases with higher RF frequencies (and MW amplitude), suggesting that possible heating effects may play a role. This might cause instabilities in the driving amplitude and thus lead to line broadening (see Supplementary Note 5 in the Supplementary Information for more details). Overall, while the XY8-*N* protocol exhibits higher sensitivity compared to the spinlock, it is not suitable for detection above 10 MHz, whereas the CDD scheme is only limited by the achievable MW power which in our case corresponds to a maximum sensing frequency of ~85 MHz.

### RF sensing beyond NV-decoherence time constraints

**Fig. 3:**
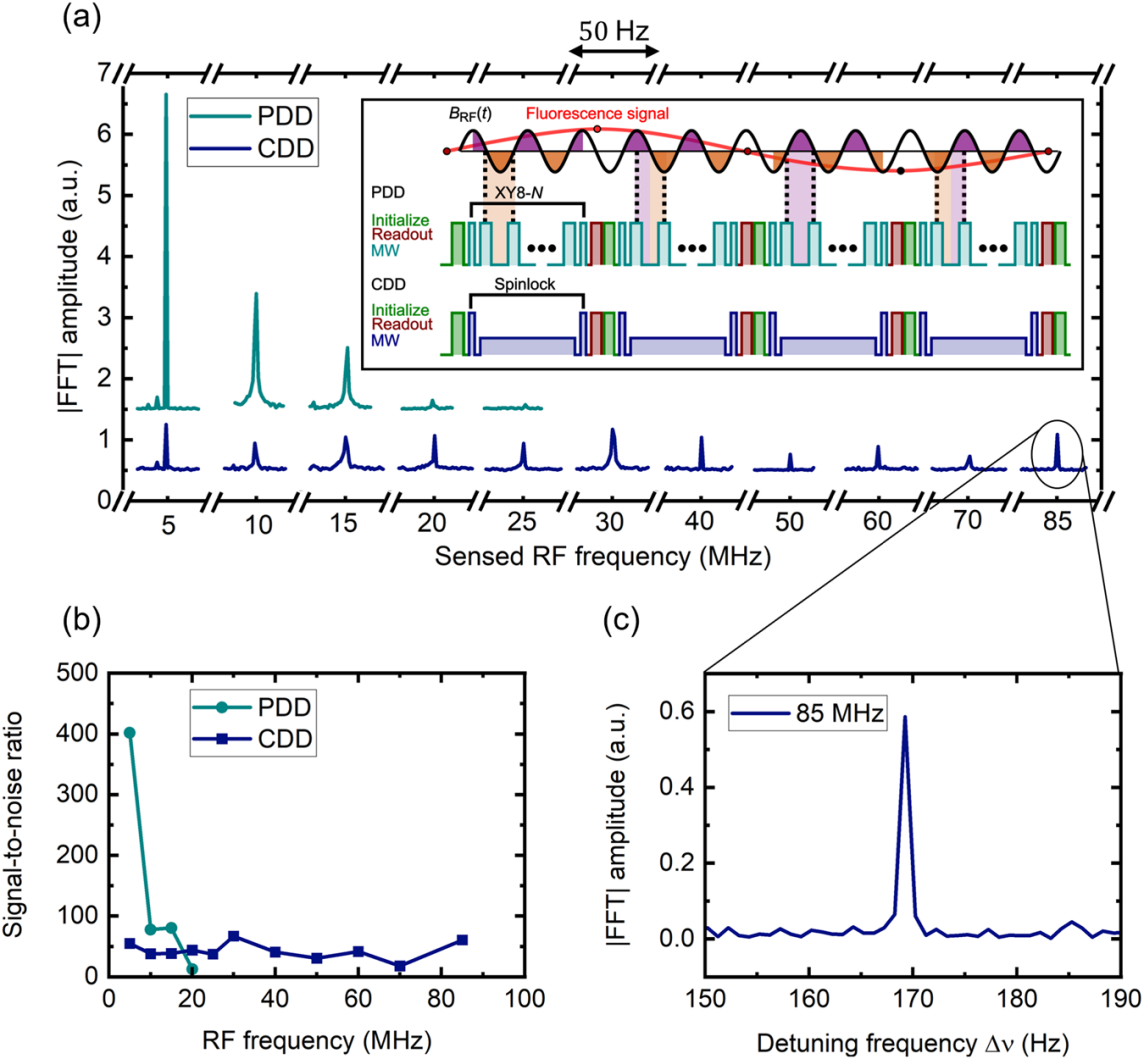
Overcoming limits on frequency resolution by coherently averaged synchronized readout (CASR). **a** PDD (XY8-*N*) and CDD (spinlock) subsequences are implemented in the CASR scheme, as depicted in the inset. The time-domain fluorescence oscillations (schematically illustrated in the inset) are recorded for each RF sensing frequency up to ~85 MHz (in case of CDD-CASR), and subsequently Fourier transformed, giving rise to the peaks at the detuning frequency Δ*ν*. Notice that the difference between two neighboring *x*-axis breaks is 50 Hz as indicated above the figure. **b** SNR of the CASR curves in (**a**). **c** Zoom-in of the CDD-CASR at an RF sensing frequency of 85 MHz for a better visualization.

The aforementioned pulse sequences are limited in the achievable spectral resolution by the maximum sensing time which is set by their respective decoherence times (*T*_2_ for XY8-*N* and *T*_1*ρ*_ for SL) or by technical contraints (such as MW inhomogeneity in CDD). To overcome this limitation, heterodyne^47,48^ or Coherently Averaged Synchronized Readout (CASR)^7^ schemes have been developed, as illustrated in Fig. 3a.

In a typical CASR experiment, consecutive PDD sequences are set on resonance with the sensing RF frequency by adjusting the corresponding *τ* spacing according to the matching condition mentioned above. Then, the sequences are synchronized with the sensing RF signal and configured to be sensitive to the signal’s phase ^26^(see Materials). This synchronization is achieved by setting the total duration of the individual PDD sequences to a multiple of the pulse spacing *τ*. A slight deviation of the RF frequency *ν*_RF_ with respect to the pulse sequence: *ν*_PDD_ = 1/(4*τ*) results in different RF phases being recorded by each individual PDD sequence. As a consequence, the accumulated phases *θ*, indicated by the violet and orange areas in the inset of Fig. 3a, differ for each subsequence. Thus, the fluorescence signal (red dots in Fig. 3a, inset) oscillates at the difference frequency Δ*ν* = ∣*ν*_PDD_ − *ν*_RF_∣ which can be Fourier transformed in order to obtain the corresponding frequency spectrum. Since the sensing time is not limited by the coherence time, the spectral resolution can be arbitrarily improved, only being limited by the clock stability of the experimental setup^7,47,48^.

Until now, this method has been implemented exclusively using PDD protocols, resulting in the same limitation regarding the maximum detectable frequency *ν*_RF_. Our approach exploits the phase sensitivity of the spinlock scheme. By satisfying the condition *ν*_RF_ = *ν*_CDD_ = *Ω*_SL_/(2*π*) and introducing a small deviation between the pulse sequence and the RF signal synchronization, a novel CDD-CASR experiment with a wider frequency detection range is enabled. In the experiments shown in Fig. 3a, we compare the Fourier transformed signal obtained by PDD-CASR using the XY8-*N* protocol with the CDD-CASR at different RF sensing frequencies. The detuning frequencies Δ*ν* were between 150–200 Hz. A detailed summary of experimental parameters can be found in the Methods part. As in the previous result, at a sensing frequency of ~5 MHz, the PDD-CASR exhibits a significantly higher SNR (see Fig. 3b) than the CDD-CASR. However, it rapidly declines for increasing RF frequencies whereas the peak heights of the CDD-CASR measurements remain fairly constant over all tested frequencies, enabling sensing of RF fields up to ~85 MHz (see Fig. 3c) with high-frequency resolution. Although approximately eight times less sensitive than PDD at sensing frequencies as low as ~5 MHz (see Fig. 3b), CDD-CASR demonstrates the ability to achieve sensitivities comparable to its counterpart at higher frequencies of around ~10–15 MHz. Importantly, it consistently performs well even at higher frequencies, proving to be a reliable method in a CASR protocol, with capabilities reaching up to ~80–90 MHz.

## Discussion

Our results highlight the CDD scheme as a complementary method to the well-established PDD for RF sensing, significantly extending the frequency detection range by an order of magnitude. The XY8-*N* PDD sequence proves, in our case, effective for sensing low RF frequencies (a few MHz) until it becomes less effective above 5 MHz. Beyond this threshold, the spinlock protocol demonstrates greater suitability due to its capability of sensing higher frequencies. However, the CDD scheme presents challenges that require further improvements in the future. Firstly, while it enables the detection of frequencies otherwise inaccessible, the method’s sensitivity is around three to eight times lower than the established PDD approach when detecting frequencies as low as a few MHz (see Fig. 3b). Since the theoretical sensitivity of the two methods is similar, the reduced CDD performance can be attributed to current experimental limitations, such as spatial inhomogeneities of the driving MW field and heating effects, both resulting in a broadening of the signal linewidth (see Fig. 2c). In particular, heating effects caused by high power and prolonged MW spinlock pulses, as detailed in Supplementary Note 5 of the Supplementary Information, likely represents the main limitation to overcome. It possibly contributes to two effects: (1) the instability of the microwave cavity resonance, which leads to a broader distribution of MW fields (*B*_1_) on the experimental scale and subsequently widens the signal linewidth; (2) an increase of the diamond temperature itself, causing a shift in the NV resonance and degrading the performance of the sensing protocol. These issues can be effectively addressed through strategies for active cooling and a more careful design of the setup, incorporating a diamond sensor with a geometry precisely tailored to the resonant cavity to minimize the contribution from the MW *E*-fields. Furthermore, solving the heating problems would allow us to take advantage of the *T*_1*ρ*_ time, which is typically much longer than the *T*_2_ time limiting the PDD counterpart, resulting in improved sensitivity^45,46,49^. Finally, methods for increased robustness of manipulating NV-dressed states need to be explored in this direction, including techniques based on continuous concatenated dynamical decoupling (CCDD) and double-drive experiments^60–66^. Reducing the size of the MW structure for smaller spin ensembles down to the single spin level would alleviate the heating problems and reduce the technical complexity of implementation.

In summary, the integration of Continuous Dynamical Decoupling (CDD) detection protocols with microwave (MW) resonators for spin ensembles extends the frequency detection range by an order of magnitude. While some technical challenges remain, in particular the optimal dissipation of heat generated by the high-power MW drive, these are primarily engineering tasks that are likely to be solved in the near future with more refined instrumental strategies. This improvement provides a valuable toolkit for quantum sensing based NMR, especially in high magnetic bias fields.

## Methods

### NV ensemble sensor

For the experiments, a 1 mm × 1 mm × 0.5 mm electronic grade diamond, provided by Element Six Ltd (Didcot, United Kingdom), was used. A nitrogen-doped layer was overgrown using chemical vapor deposition (CVD) by Applied Diamond, Inc. (Wilmington, DE, United States). The nitrogen concentration after the growth is estimated to be ~10 ppm and the layer thickness is approximately 10 μm. The diamond was irradiated with electrons with an energy of 1 MeV and a fluence of 3 × 10^18^ cm^−2^ to create vacancies in the lattice. Subsequently, the diamond was annealed in vacuum for 12 hours at a temperature of 800°C to form NV centers.

### Experimental setup

As depicted in Fig. 1c, the diamond is placed into a dielectric microwave resonator operating in the X-band regime at ~9.4 GHz. For an efficient light collection an optical glass hemisphere (TECHSPEC N-BK7, Edmund Optics) is glued on top of the diamond. After positioning this assembly inside an electromagnet (model 5405, GMW Associates) which is oriented such that the magnetic field *B*_0_ aligns with one of the four NV axes, the NV center’s $\left\vert 0\right\rangle \leftrightarrow \left\vert +1\right\rangle$ transition frequency is tuned to the resonator eigenfrequency by adjusting the current through the electromagnet to a corresponding field of ~230 mT. For initialization and readout of the NV spin state, a laser (Opus 532, Novanta photonics) with a power of approximately 200 mW is used. Pulsing of the laser is enabled by an acousto-optic modulator (3250-220, Gooch and Housego) with a pulse duration of 1.1 ms. This long pulse duration is chosen to maximize the dead time between CDD sequences in order to mitigate possible resonator heating effects (see Supplementary Note 5 in the Supplementary Information). After passing a *λ*/2 waveplate which sets the polarization for optimal NV excitation, the laser light is focused by a lens with a focal length of 75 mm down to an estimated spot diameter of ~50 μm on the NV diamond under a total reflection geometry. The fluorescence light is collected and collimated by a condenser lens (ACL25416U-B, Thorlabs) placed above the diamond and subsequently led through a long-pass filter (Edge Basic 647 Long Wave Pass, Semrock) and focused on a photodiode (PDA100A2, Thorlabs) by another condenser lens of the same model. The photodiode is then read out by a data acquisition unit (USB-6281 DAQ, National Instruments) which is connected to a computer.

An arbitrary waveform generator (AWG 5202, Tektronix) synchronizes the experiment and generates the microwave pulse sequences. An additional MW source (SMB 100A, Rohde&Schwarz) acts as a local oscillator whose signal (8.9 GHz) is mixed with that of the AWG (0.5 GHz) via an IQ-mixer (MMIQ-0218LXPC, Marki Microwave) to achieve the desired ~9.4 GHz output frequency. After pre-amplification (ZX60-153LN-S+, Mini-Circuits), the MW signal reaches the ~60 W main amplifier (RFLUPA08G11GA, RF-Lambda) and thereafter the MW resonator.

The RF fields to be sensed in the experiments are delivered by placing a self-assembled copper coil near the diamond. The coil is soldered on a BNC cable and connected to an amplifier (LZY-22+, Mini-Circuits) and RF source (DG4162, RIGOL). To lock the RF signal to the pulse sequence in the CASR experiments, the RF source is synchronized with the AWG. In order for the coil to produce approximately the same magnetic field output for each sensing frequency, the RF amplitude was calibrated before the experiments, as described in Supplementary Note 6 in the Supplementary Information.

### X-band microwave resonator

The resonator was constructed from high permittivity ceramics (*ϵ* = 80) and was machined into the shape of a ring with an outer diameter of 4.8 mm, an inner diameter of 2 mm, and a height of 1.55 mm. It is excited by a microstrip line positioned beneath it. The desirable resonance mode for our application is TE_01*δ*_ (see Fig. 2 in Supplementary Note 2 of the Supplementary Information), which produces a high and homogeneous driving field ${\hat{B}}_{1}$ in the center of the structure. For this mode, the resonance frequency is approximately 9.4 GHz, and the loaded quality factor *Q* of the resonator, when critically coupled, is found to be ~700.

### XY8-*N* and spinlock protocols

The XY8-*N* experiments are conducted in accordance with the following scheme, ${(\pi /2)}_{y}\,{\left[{{[-\tau -{(\pi )}_{\phi }-\tau]}_{8}}\right]}_{N}\,{(\pi /2)}_{y}$, where the (relative) phases of the *π*-pulses are chosen such that the rotational axes alternate between the *x*- and *y*-axis: *ϕ* = [*x*-*y*-*x*-*y*-*y*-*x*-*y*-*x*] (see inset of Fig. 2). The durations of the *π*/2 and *π* pulses are 3 ns and 6 ns, respectively (see Supplementary Note 3 in the Supplementary Information). The first *π*/2 pulse and the last projection pulse are in phase (*y*-axis as rotational axis), enabling RF-phase insensitive variance detection (quadratic detection) which is suitable for sensing incoherent signals (see Supplementary Information of ref. ^46^ for more details). For noise canceling purposes, a second referencing sequence is implemented, only differing in the relative phase of the last projection pulse: (*π*/2)_(−*y*)_. Each data point is then obtained by normalizing the difference between measurement and reference readouts to their sum and by averaging them 2000 times.

In this experiment, the RF source generated a non-synchronized signal by keeping it unlocked in regards to the pulse sequence so that each repetition of the sequence results in a different RF phase being recorded. For each sensing frequency, the corresponding spacing between *π* pulses is kept constant while the RF frequency is swept and the fluorescence signal is recorded. The phase accumulation time *t*_*s*_, that is the timeframe between initial and last *π*/2 pulse is 5 μs for all experiments. This, together with the dead time of 1.1 ms set by the laser pulse, results in a duty cycle of ~0.5%. Such a low duty cycle is chosen to reduce heating effects of the resonator, which could negatively affect the performance of the spinlock experiment (see Supplementary Note 5 in the Supplementary Information).

The spinlock experiment in variance detection mode consists of the following pulsing scheme: (*π*/2)_*x*_ (Spinlock)_*y*_ (*π*/2)_*x*_, as illustrated in the inset of Fig. 2a. Referencing is achieved by changing the relative phase of the last projection pulse to (*π*/2)_(−*x*)_. The experiments are conducted in the same manner as the XY8-*N* measurements with identical phase accumulation times and number of averages. For every sensing frequency, the amplitude *Ω*_SL_ is set accordingly, and the RF frequency is swept.

### CASR protocol

The CASR protocol consists of concatenated DD subsequences, whereby the total duration of each subsequence is set to a multiple of the pulse spacing *τ*. The RF frequency to be sensed slightly deviates from the matching condition, such that the obtained contrast oscillates with the detuning frequency Δ*ν* = ∣*ν*_DD_ − *ν*_RF_∣. In this experiment, detuning frequencies between 150 and 200 Hz are chosen. In case of the PDD sequence, the spacing *τ* is set to a desired matching frequency *ν*_PDD_ = 1/(4*τ*) (e.g. 5 MHz) and the RF signal is adjusted according to *ν*_RF_ = *ν*_PDD_ + Δ*ν*. For the CDD protocol, a sweep of the spinlock amplitude *Ω*_SL_ is carried out while keeping the RF frequency constant at a chosen matching frequency. After identifying and setting the SL amplitude to the desired measurement frequency *ν*_RF_ = *ν*_CDD_ = *Ω*_SL_/(2*π*) at the fluorescence dip, a small deviation between the pulse sequence and RF synchronization is introduced, leading to the oscillating contrast in the time-domain.

The CASR protocol requires sensitivity to the phase of the coherent RF signal, hence the last *π*/2 projection pulse of the subsequences differ from the previous experiment: (*π*/2)_*x*_ and (*π*/2)_*y*_ for the XY8-*N* and spinlock subsequences, respectively. This sets the scheme to a slope detection mode (linear detection) which is sensitive to coherent signals^46^. The phase accumulation time of the subsequences is kept at 5 μs and the total duration of the measurement is 1 s. The time signal is Fourier transformed and the resulting frequency spectrum ∣FFT∣ is plotted in Fig. 3.

## Supplementary information

Supplementary Information to: Extending Radiowave Frequency Detection Range with Dressed States of Solid-State Spin Ensembles
