# Supplementary material for: Extending radiowave frequency detection range with dressed states of solid-state spin ensembles
Source: npj Quantum Inf. 2024 Oct 26;10(1):103. doi: 10.1038/s41534-024-00891-0 (PMC11512814; doi:10.1038/s41534-024-00891-0)
Supplement: Supplementary file 1 — Supplementary Information to: Extending Radiowave Frequency Detection Range with Dressed States of Solid-State Spin Ensembles [file 41534_2024_891_MOESM1_ESM.pdf]

Supplementary Information to “Extending Radiowave Frequency Detection Range with Dressed States of Solid-State Spin Ensembles”

Jens C. Hermann[1,2†], Roberto Rizzato[1,2*†], Fleming Bruckmaier[1,3], Robin D. Allert[1], Aharon Blank[4] and Dominik B. Bucher[1,2*]

[1]Technical University of Munich, TUM School of Natural Sciences, Department of Chemistry, Lichtenbergstraße 4, Garching bei München, 85748, Germany.
[2]Munich Center for Quantum Science and Technology (MCQST), Schellingstr. 4, München, 80799, Germany.
[3]QuantumDiamonds GmbH, Friedenstr. 6, München, 81671, Germany.
[4]Schulich Faculty of Chemistry, Technion - Israel Institute of Technology , Haifa, 32000, Israel.

*Corresponding author(s). E-mail(s): roberto.rizzato@tum.de; dominik.bucher@tum.de;
†These authors contributed equally to this work.

Supplementary Note 1: Direct observation of spinlock phase accumulation dynamics

As discussed in the main text, the phase θ_{SL} accumulated during a spinlock pulse at the matching condition $\Omega_{\text{SL}} = 2\pi\nu_{\text{RF}}$ is given by $\theta_{\text{SL}} = \frac{1}{2}\gamma\hat{B}_{\text{RF}}t_s$ with the gyromagnetic ratio γ, the magnetic RF field amplitude $\hat{B}_{\text{RF}}$ and the spinlock duration t_s [1]. In contrast to PDD sequences, where phase is accumulated on the xy-plane of the Bloch sphere in the first rotating frame, the spin vector moves in a spiral motion from the $(-y)$ towards the $(+y)$ axis during the spinlock pulse, as indicated by the black arrow in Supplementary Figure 1 (a), and then back to the $(-y)$ axis (blue arrow).
Experimentally, the spin dynamics can be probed by leaving out the last $\pi/2$ projection pulse of the typical CDD sequence shown in the inset of Figure 2 (a) of the main text. In Supplementary Figure 1 (b), the experimental result for a spinlock amplitude of $\Omega_{\text{SL}} = 2\pi \cdot 5\,\text{MHz}$ is shown, exhibiting fast oscillations at the Rabi frequency (5 MHz) for a full evolution from the $(-y)$ axis towards the $(+y)$ axis (black) and back (blue).

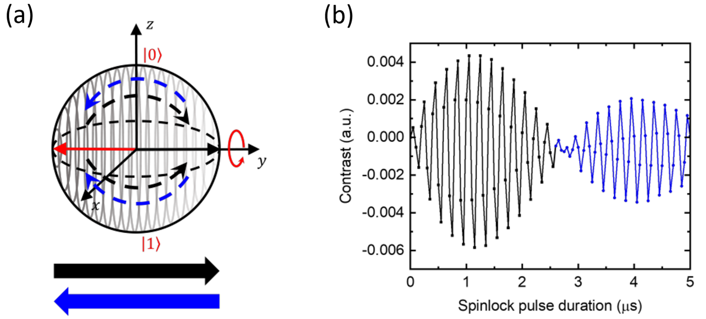

Supplementary Figure 1: **CDD spin dynamics.** (a) Schematic depiction of the spiraling motion in the first rotating frame during spinlock phase accumulation. (b) Direct observation of the spiraling dynamics by omitting the last $\pi/2$ projection pulse.

Supplementary Note 2: Microwave resonator design

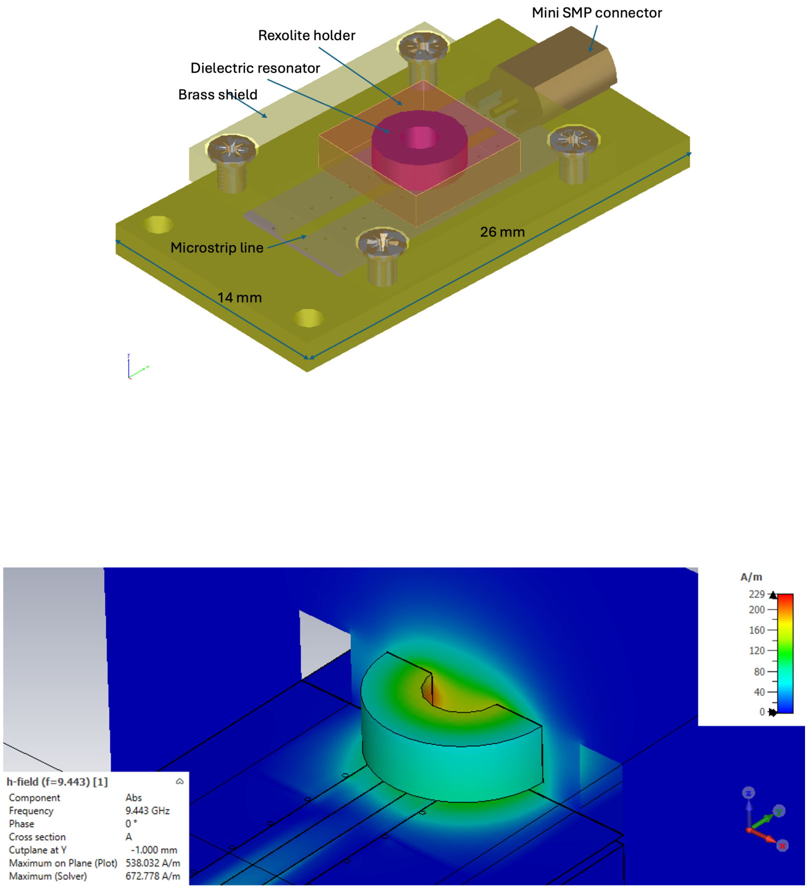

Supplementary Figure 2: **Resonator design.**

Supplementary Note 3: Rabi experiment

To determine the π and $\pi/2$ pulse durations, a Rabi experiment was performed whereby the MW pulse duration was swept at the maximum MW power. Supplementary Figure 3 displays the measured Rabi-oscillations up to a pulse duration of 200 ns (3 (a)) and up to 20 ns (3 (b)). From this experiment, $t_{\pi/2} \approx 3$ ns and $t_{\pi} \approx 6$ ns were determined, corresponding to a Rabi

frequency of about 80 MHz with a maximum contrast of approximately 4%. Furthermore, Supplementary Figure 3 (a) exhibits almost no decay in contrast over a timeframe of 200 ns, indicating a homogeneous MW field over the laser spot (estimated spot diameter of $\sim 50\,\mu$m) provided by the MW resonator.

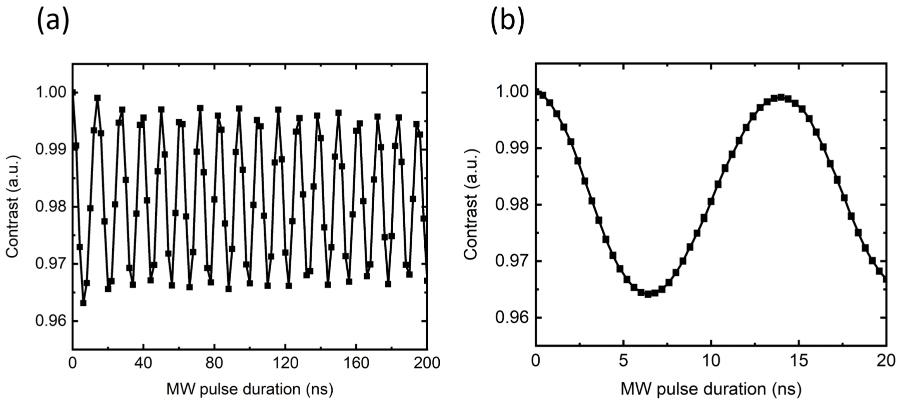

Supplementary Figure 3: **Rabi experiment.** Measured Rabi oscillations up to a MW pulse duration of 200 ns (a) and 20 ns (b).

Supplementary Note 4: Resonator ringdown time

The microwave pulses used to drive the NV spin states are not perfectly rectangularly shaped, but they exhibit a significant ringdown decay time due to the microwave resonator's Q-factor of approx. 720, which was measured with a vector network analyzer. The Q-factor together with the resonance frequency f_0 at 9.4 GHz correspond to an estimated decay time t_d of ≈ 25 ns according to [2, 3]

$$t_d = \frac{Q}{\pi f_0}\,. \tag{1}$$

This has been verified experimentally by recording the (downmixed) reflected signal behind the resonator with an oscilloscope. Supplementary Figure 4 (a) shows an incoming π pulse (marked area 1) with a set pulse duration of ≈ 6 ns and its reflection behind the resonator (area 2). In contrast to the incoming pulse, the reflected signal displays a considerably long ringdown time which is in good agreement with the aforementioned calculated value.
As a consequence, an overlap of pulses for sensing frequencies at and above 10 MHz occurs: As shown in Supplementary Figure 4 (b), the spacing τ between the first $\pi/2$ and π pulse (measured from the center of the pulses) can be expressed as follows:

$$\tau = \frac{3}{4}t_\pi + t_d + \Delta t \tag{2}$$

where t_π is the π-pulse duration and Δt the separation between the pulses taking into account the decay time t_d. For $\Delta t < 0$, the tail of the $\pi/2$-pulse overlaps with the subsequent π-pulse, negatively affecting the sensor's capability to manipulate the spin qubit accurately. The minimum separation without overlap is therefore $\Delta t = 0$, corresponding to a spacing of $\tau_{\text{overlap}} = \frac{3}{4}t_\pi + t_d \approx 30\,\text{ns}$ for $t_\pi = 6\,\text{ns}$ and $t_d = 25\,\text{ns}$. The minimal sensing frequency at which the pulses overlap is given by the matching condition $\nu_{\text{overlap}} = 1/(4\tau_{\text{overlap}}) \approx 8\,\text{MHz}$ which matches the observations in the main experiments (see Results in the main text).

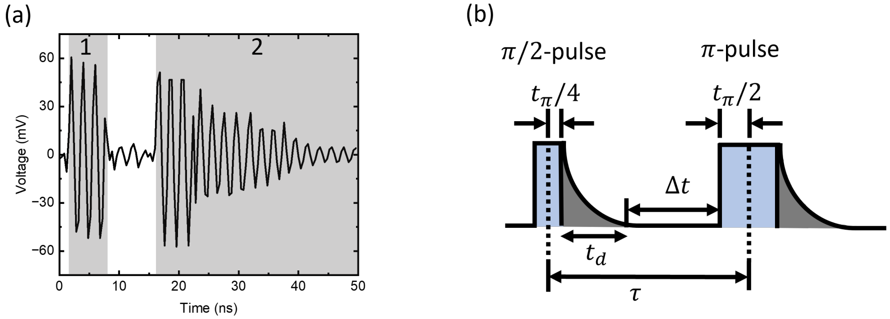

Supplementary Figure 4: **Microwave pulse characterization.** (a) The incoming π-pulse (marked area 1) is reflected behind the resonator (area 2) which exhibits a significantly long characteristic decay time. (b) Illustration of the relationship between pulse spacing τ, π-pulse duration t_π, decay time t_d and pulse separation Δt.

Supplementary Note 5: Evidence for MW resonator heating effects

The microwave resonator, connected to a $\sim 60\,\text{W}$ amplifier, generates strong and homogeneous MW fields required for high-frequency NV sensing. To investigate possible heating effects due to the strong MW power used, the temperature on the resonator next to the diamond position was probed using a temperature sensor (Thorlabs TSP01). The chosen MW power was at 60% of its maximum value and the tested duty cycles were 20% (100 µs MW duration and 400 µs dead time) and 1% (5 µs MW duration and 400 µs dead time). The duty cycle in the experiments described in the main text is $\approx 0.5\%$ (5 µs MW duration and 1.1 ms dead time).

As evident from Supplementary Figure 5, the recorded temperature rises from just above 20°C to ≈ 70°C in case of a duty cycle of 20% and to ≈ 30°C for the 1% duty cycle, indicating significant heating effects which might lead to instabilities that may result in line broadening.

The results displayed in Supplementary Figure 2 of the main text have been

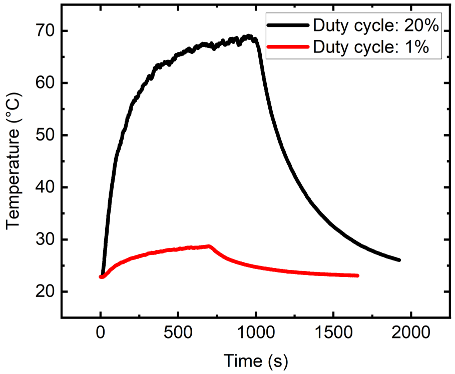

Supplementary Figure 5: **Resonator temperature for two different duty cycles.** The black curve corresponds to a set duty cycle of 20% whereas the red curve results from a duty cycle of 1%.

reproduced with a longer spinlock pulse duration of $t_s = 20\,\mu s$ and dead time of 1.5 ms corresponding to a duty cycle of $\approx 1\%$. The applied RF field amplitude (see Supplementary Note 4) has been scaled such that the accumulated phase during the spinlock duration is the same for both experiments ($t_s = 5\,\mu s$ and $t_s = 20\,\mu s$). The comparison in Supplementary Figure 6, exhibits a considerably lower contrast and lower achievable maximum RF sensing frequency for a higher duty cycle. As already mentioned, suspected heating effects may deteriorate the CDD sensing capabilities.

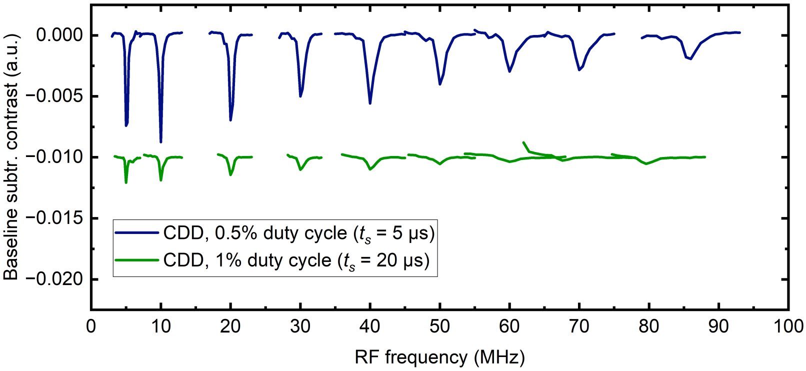

Supplementary Figure 6: **Frequency response of the CDD detection scheme with two differing duty cycles.** The blue curve (from Figure 2 in the main text) corresponds to an active sensor time t_s of $5\,\mu s$ and a dead time of 1.1 ms (duty cycle of $\approx 0.5\%$) and the green curve to a spinlock duration of $20\,\mu s$ a dead time of 1.5 ms (duty cycle of $\approx 1\%$).

Supplementary Note 6: Calibration of the RF amplitude $\hat{B}_{\mathrm{RF}}$

The RF signal sensed by the NV detector is provided by a self-assembled coil near the diamond position. The impedance of the RF coil is frequency dependent, thus, the output voltage of the RF signal generator was calibrated such that the sensed RF amplitude $\hat{B}_{\mathrm{RF}}$ is approximately the same for each measurement frequency. This was achieved by placing a pick-up coil (Beehive Electronics 100A) near the RF coil and by reading out the pick-up coil's induced voltage amplitude via an oscilloscope at a constant RF signal source voltage for each applied frequency. Supplementary Figure 7 shows the magnetic field output of the RF coil in dependence of the RF frequency at the position of the pick-up coil. From this, the voltage of the RF source was adjusted such that the amplitude $\hat{B}_{\mathrm{RF}}$ is constant over all applied RF frequencies.

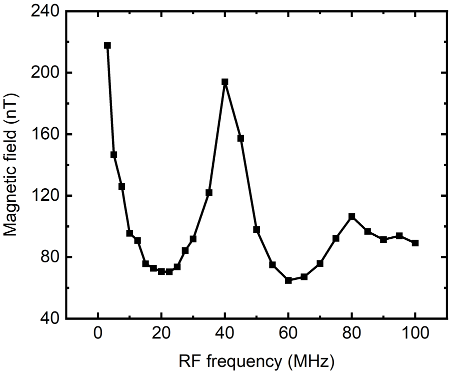

Supplementary Figure 7: **Calibration of the RF amplitude $\hat{B}_{\mathrm{RF}}$.** Due to the frequency dependency of the RF coil's impedance, the amplitude of the RF source was adjusted for a constant magnetic field output for each sensing frequency in the main experiments.

References

[1] Rizzato, R., Schalk, M., Mohr, S., Hermann, J.C., Leibold, J.P., Bruckmaier, F., Salvitti, G., Qian, C., Ji, P., Astakhov, G.V., Kentsch, U., Helm, M., Stier, A.V., Finley, J.J., Bucher, D.B.: Extending the coherence of spin defects in hBN enables advanced qubit control and quantum sensing. Nature Communications **14**(1), 5089 (2023). https://doi.org/10.1038/s41467-023-40473-w

[2] Jackson, J.D.: Classical Electrodynamics, 3rd edn. John Wiley & Sons, Hoboken, NJ (1998)

[3] Heintze, J., Bock, P.: Lehrbuch zur Experimentalphysik Band 1: Mechanik. Springer Berlin Heidelberg, Berlin, Heidelberg (2014). https://doi.org/10.1007/978-3-642-41210-3
